# Atomic force microscopy-based microrheology reveals significant differences in the viscoelastic response between malign and benign cell lines

**DOI:** 10.1098/rsob.140046

**Published:** 2014-05-21

**Authors:** Jan Rother, Helen Nöding, Ingo Mey, Andreas Janshoff

**Affiliations:** 1 Institute of Physical Chemistry, Tammannstrasse 6, 37077 Göttingen, Germany; 2 Institute of Organic and Biomolecular Chemistry, Tammannstrasse 2, 37077 Göttingen, Germany

**Keywords:** microrheology, atomic force microscopy, cancer, viscoelasticity

## Abstract

Mechanical phenotyping of cells by atomic force microscopy (AFM) was proposed as a novel tool in cancer cell research as cancer cells undergo massive structural changes, comprising remodelling of the cytoskeleton and changes of their adhesive properties. In this work, we focused on the mechanical properties of human breast cell lines with different metastatic potential by AFM-based microrheology experiments. Using this technique, we are not only able to quantify the mechanical properties of living cells in the context of malignancy, but we also obtain a descriptor, namely the loss tangent, which provides model-independent information about the metastatic potential of the cell line. Including also other cell lines from different organs shows that the loss tangent (*G″*/*G′*) increases generally with the metastatic potential from MCF-10A representing benign cells to highly malignant MDA-MB-231 cells.

## 2. Introduction

In cancer biology, the conversion from non-tumorigenic cells into metastatic ones is of pivotal interest, especially since formation of metastases poses the biggest threat for humans diagnosed with cancer. During the progression from a benign tumour to a malign neoplasm, the cells undergo many changes on the molecular level. In cancer, not only is the cell cycle machinery out of control—other regulatory processes like cellular adhesion and migration are also affected [1]. This becomes obvious while looking at key steps in the formation of metastasis, which includes invasion into surrounding tissue, and intra- and extravasation into lymphatic or blood vessels. These steps involve dramatic changes in the organization of the cellular cytoskeleton. In particular, changes in the expression of proteins, which are associated with regulation and dynamics of the actin cytoskeleton, play an important role in many types of cancer [2]. Therefore, it is conceivable that, apart from structural changes, alterations of mechanical and dynamical properties also take place during tumour progression, providing a signature of malignancy. To date, there are several studies available that report on measurements of rigidity of cancer cells in comparison with benign cells. Most studies find that cancer cells are significantly softer than normal cells [3]. Recently, Agus *et al*. [4] published a thorough study comparing the two cell lines MCF-10A and MDA-MB-231. MCF-10A is an immortal but non-tumorigenic human breast cell line, whereas MDA-MB-231 cells represent the malignant state [4]. It was found that the metastatic MDA-MB-231 cells were fourfold more elastic (i.e. softer) than the non-tumorigenic MCF-10A cells. This is understandable as softer cells would more easily penetrate tissues and the extracellular matrix, and hence increase the invasiveness of tumour cells. By contrast, there also exist studies that report on a stiffening of cancer cells compared with benign cells, suggesting that tumour progression can have diverging effects on cellular elasticity [5,6]. Measurements of these mechanical changes are frequently lumped into a single universal parameter: Young's modulus. The suitability of Young's modulus to describe the malignancy of cells is, however, a matter of debate. It is therefore of great interest to identify reliable mechanical descriptors that predict faithfully the malignancy of cells. Here, rheological approaches might provide the key data since they are intimately linked to the activity of motor proteins in cells [7]. Numerous techniques have been used to quantify the mechanical properties of cells, including magnetic bead twisting, micropipette aspiration, particle tracking, optical tweezers, microplate rheology and atomic force microscopy (AFM) [8]. Among these methods, AFM is the technique with the highest spatial resolution and the largest force range, covering piconewton to micronewton by using a small tip attached to a flexible cantilever [9]. Atomic force microscopy has widely been used to determine elastic properties of different samples, predominantly applying contact models such as the Hertz or JKR model [10]. However, the occurrence of a hysteresis between indentation and retraction curve in force–distance measurements indicates that cells do not exhibit a purely elastic behaviour suggesting that it would be useful to describe the mechanical properties of cells in the context of time-dependent rheology. To obtain information about the frequency-dependent viscoelastic properties of samples using the AFM, Shroff *et al*. [11] developed a method that uses small-amplitude oscillations of the AFM cantilever in contact with the cell body. They were able to determine the apparent dynamic modulus, the ratio between applied force and sample indentation, of single cardiomyocytes during contraction. Quantitative models to compute the complex shear modulus G*, which accounts for the contact area as a function of the indentation depth, were developed by several groups considering either a spherical or pyramidal indenter geometry to probe the sample [12–14]. Microrheological studies of adherent cells carried out by a large variety of techniques suggest that the cytoskeleton resembles the viscoelastic nature of soft glassy materials over a wide frequency range due to its general scaling behaviour [12]. This interpretation implies that non-equilibrium interactions on the meso scale expressed by the cytoskeletal lattice of living cells are central to understand their rheological behaviour associated with fundamental functions of the cell originating from cytoskeletal dynamics. Those functions comprise embryonic development, wound healing, cell migration, metastasis and invasion [15].

With the focus on malignancy, we investigated nine cell lines (NIH 3T3 fibroblasts, MDCK-II, NMuMG, A549, SW13, MCF-7, MCF-10A, MDA-MB-231 and CaKi-1 cells) from four organs that possess different metastatic potentials. Among them, three (MCF-10A, MCF-7 and MDA-MB-231) are human breast cell lines, in which MCF-10A represents the benign control, MCF-7 displays a moderate metastatic potential and MDA-MB-231 is considered highly malignant. We find that cells from the same organ but with higher malignancy are generally softer than benign cells, which is expressed in a lower real part of the complex shear modulus. However, when comparing cells of different metastatic potential from different organs, there is no clear trend in rigidity. Nonetheless, considering malignant cells that originate from the same organ and organism, such as MCF-7 and MDA-MB-231 cells, we could confirm that these cells are softer than benign MCF-10A cells. Interestingly, however, all cancer cell lines displayed a higher loss tangent (*η* = *G″/G′*) at high oscillation frequencies compared with the benign cell lines, regardless of their origin. The loss tangent is inherently model-independent and conveys information as to whether a cell is rather solid or fluid at a given excitation frequency. Our measurements suggest that cancer cells are generally more fluid-like than epithelial cells or fibroblast, accounting for the migratory behaviour of the malign cells.

## 3. Measurement and data processing

**Figure 1.**
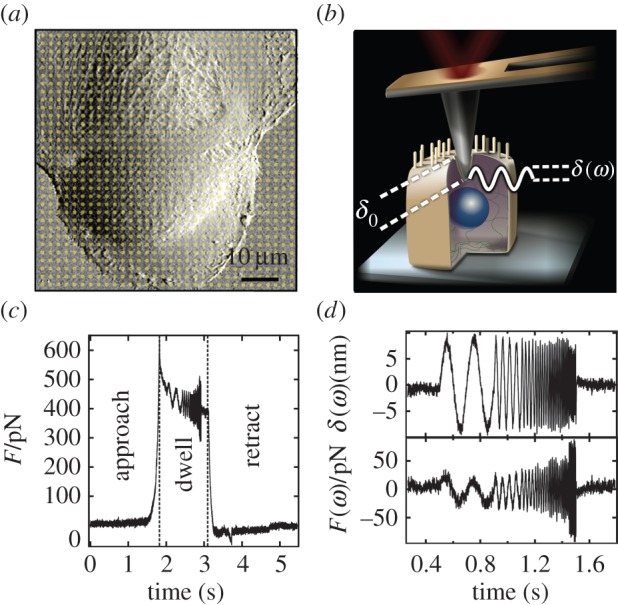
(*a*) Illustration of force mapping on cells. A deflection AFM image (contact mode) of subconfluent NMuMG cells is shown. During force mapping a force–distance curve is taken at every yellow spot. (*b*) Schematic drawing of the experiment: the cantilever oscillates around the indentation depth *δ*_0_ with an amplitude *δ* at the frequency *ω*. (*c*) Time course of force during the measurement of a force–distance curve. When the cantilever gets into contact with the sample, the force increases rapidly until the present trigger point is reached. During dwell in contact, the cantilever is excited to sinusoidal oscillations with frequencies from 5 to 200 Hz. Afterwards, the cantilever is retracted and the procedure repeated at a different position. (*d*) Indentation oscillation *δ*(*ω*) with frequencies from 5 to 200 Hz around the indentation depth *δ*_0_ and corresponding force signal *F*(*ω*) after detrending.

Experiments were carried out using a commercially available MFP-3D AFM (Asylum Research, Santa Barbara, CA) placed on an inverted microscope (IX51, Olympus, Tokyo, Japan). After calibration of the spring constant of the cantilever (MLCT, Veeco, C-lever, nominal spring constant *k* = 0.01 N m^−1^) using thermal noise spectra and determination of the optical lever sensitivity, cells were imaged in contact mode. Subsequently, we used the built-in force map mode of the AFM to measure force–distance curves providing information about the local mechanical response of the cells on the previously imaged area. A typical example of subconfluent NMuMG cells is shown in figure 1*a*. Every spot in this particular AFM image corresponds to a single force curve in the force map measurement. A schematic drawing of the experimental set-up is shown in figure 1*b*. Figure 1*c* depicts the time course of the force recorded during the measurement of a force–distance curve modulated with a sinusoidal oscillation.

In general, the method is based on the following idea; if a cantilever is placed on a hard or an elastic surface and is excited harmonically but off-resonance at its basis, the deflection signal and the sinusoidal excitation signal are in phase. Placing the cantilever into a viscous environment and exciting it in the same way, it exhibits its maximum deflection when moved fastest through the fluid. This is the case at the inflection point of the sinusoidal excitation, meaning that in a viscous environment the excitation and the deflection are 90° out of phase. In summary, whether a sample behaves more like an elastic solid or more like an viscous fluid is expressed in the phase shift between the two signals, which can have values between 0° and 90°. As soon as the cantilever tip gets into contact with the sample, the force acting on the cantilever increases up to a previously chosen trigger point. After a short resting period, the cantilever is excited to an oscillatory movement *δ*(*ω*) around the given indentation depth *δ*_0_ with frequencies ranging from 5 to 200 Hz using the built-in indenter panel of the Asylum Research MFP software. During this procedure, the tip never loses contact with the cell body. Afterwards, the cantilever is retracted from the cell surface and the force decreases to zero again. Figure 1*d* shows the time course of the detrended oscillation *δ*(*ω*) around *δ*_0_ and the corresponding force response *F*(*ω*). While the amplitude of the separation variation *A_*δ*_* remains constant, the amplitude of the force response *A*_F_ increases considerably with increasing oscillation frequency. To obtain quantitative data, we adopted the mechanical model proposed by Alcaraz *et al*. [12]. The model essentially relies on Hertzian contact mechanics assuming a pyramidal indenter geometry [16]3.1
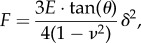
where *E* is Young's modulus, *ν* is the Poisson ratio assumed to be 0.5 and *θ* is the half opening angle of the pyramidal cantilever tip. After linearization for small amplitude oscillations according to Mahaffy and co-workers and transformation into the frequency regime to include energy dissipation, the following expression for the complex shear modulus G* using the relationship *G* = *E*/(2(1 + *υ*)) is obtained [13,17]:3.2
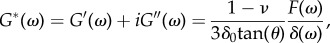
where *ω* denotes the angular frequency*.* After correction for the hydrodynamic drag acting on the oscillating cantilever and the unavoidable phase lag between excitation of the cantilever and the force response, we finally obtain the following expression for the complex shear modulus:3.3
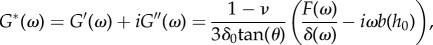
where *b*(*h*_0_) is the drag coefficient at an extrapolated tip-surface in direct contact and *i* is the complex identity. A detailed description of the data processing to correct for unwanted phase shifts can be found in the electronic supplementary material, figures S1 and S2.

## 4. Material and methods

All cell lines were cultured on glass slides (Asylum Research) at 37°C and 5% CO_2_ until confluence is reached (if possible), and were measured 1 or 2 days after seeding. When confluency was reached (except NIH 3T3, SW13 and MDA-MB231) the glass slides were mounted into the BioHeater sample stage (Asylum Research) and covered with the appropriate HEPES-buffered medium. The temperature of the BioHeater was set to 37°C throughout all measurements.

## 5. Results and discussion

**Figure 2.**
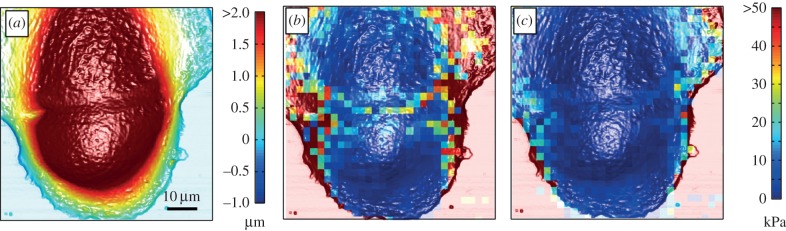
(*a*) AFM height image of subconfluent epithelial NMuMG cells (contact mode). (*b,c*) Height image overlaid with the force map data of (*b*) *G*′ and (*c*) *G*″ at an oscillation frequency of 20 Hz.

Spatially resolved microrheological data acquired for nine different cell lines were compared with respect to their malignant potential. Except for fibroblasts, all cell lines were investigated after confluence was reached. Prior to force mapping, the area of interest of each sample was imaged with the AFM to control morphology and confluency of cells. An example of subconfluent NMuMG cells that demonstrates the typical spatial resolution of the microrheological experiment using the AFM is shown in figure 2. The height image (figure 2*a*) shows two NMuMG cells in close contact to each other with maximal height of 5.5 µm (red area). In the peripheral areas of the cells, the apical membrane has a distance of a few hundred nanometres from the substrate. We assume that the cells in this image reside in a phase shortly after cytokinesis due to the lack of cell–cell contacts and the presence of a small furrow between the cells [18]. Figure 2*b* and figure 2*c* show an overlay of the height image and the corresponding storage and loss modules, respectively. The oscillation frequency of the microrheological measurement was set to 20 Hz in this representative image. Notably, at this frequency the values of *G*′ exceed those of *G*″. In general, the cells exhibit higher moduli in the peripheral areas, reaching values of more than 50 kPa for *G′* compared with only 1–5 kPa in the cells' centre. The moduli in the centre of each cell are lower compared with those in the periphery, but also with values at the interface between the two cells. The high values of *G*′ at the cell–cell interface can be explained by the presence of a stiff, contractile actomyosin ring that is necessary for the separation of the daughter cells during cytokinesis [19]. The dense network of actin filaments in direct contact with the cell membrane facilitates a higher resistivity against externally applied forces and therefore leads to higher modules.

Although figure 2 demonstrates the feasibility of the method to measure frequency-dependent mechanical parameters with high lateral resolution, the stiff underlying substrate might compromise the measurement. This effect is known as ‘bottom effect’ and lets the cells appearing stiffer, especially in regions where the cells are thin [20]. This could be a reason for the elevated values of *G*′ and *G*″ in the periphery of the cells in figure 2*b,c*. This effect can be largely ruled out since mainly confluent monolayers of the different cells lines were analysed using an indentation depth between 300 and 800 nm (500–800 nm in the case of MDA-MB-231 cells) to avoid a pseudo-stiffening of the cells.

**Figure 3.**
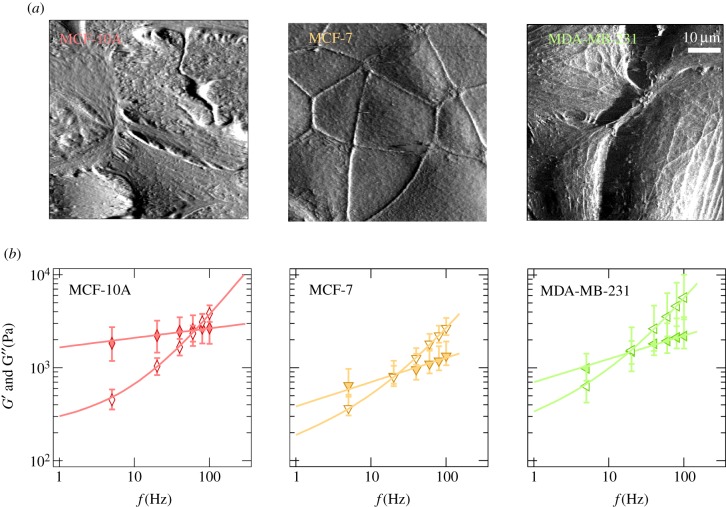
(*a*) AFM-deflection images of MCF-10A, MCF-7 and MDA-MB-231. Cells were imaged in constant force mode using pyramidal cantilever-tip geometry. (*b*) Median values of the storage modulus *G*′ (filled symbols) and loss modulus *G*″ (open symbols) as a function of oscillation frequency (two force maps, more than 10 cells). The data of the complex shear modulus were fitted using the power-law structural damping model (solid lines).

To investigate the frequency-dependent mechanical response of cancer cells compared with normal cells, we chose eight cell lines from four different organs. MDCKII cells and CaKi-1 cells originate from the kidney; SW13 are derived from the adrenal gland; NMuMG, MCF-10A, MCF-7 and MDA-MB-231 are cells from the mammary gland, and A549 cells are derived from the lung epithelial layer. MDCKII and NMuMG cells show epithelial morphology, growing as a confluent monolayer with strong intercellular junctions and a pronounced apical–basal polarity [21]. MCF-10 is an immortal non-tumorigenic epithelial cell line derived from benign breast tissue. Although the cells are immortal, they display a normal, non-cancerous phenotype. MCF-7 cells grow in a dense monolayer but also showed the ability to form metastasis in lung, liver and spleen in athymic nude mice [22]. Although forming a confluent, polarized monolayer is a characteristic of normal epithelial cells, the CaKi-1 is a representative metastatic renal cancer cell line [23]. Similarly, MDA-MB-231 cells are considered to be malignant cancer cells derived from the mammary gland, forming metastases in various organs, including lung and bones [24]. The A549 cell line originates from non-small-cell lung cancer adenocarcinoma cells and shows an epithelial-like morphology. However, in contrast to the two epithelial cell lines used in this study, the A549 cells bear the potential to form metastasis in *in vivo* models [25]. SW13 cells belong to the small-cell carcinoma and are derived from the adrenal gland [26]. Apart from the aforementioned cell lines, we also investigated fibroblasts as a paradigm for a benign mesenchymal cell as well as MCF-10A cells representing non-tumorigenic cells. Representative AFM-deflection images of all cell lines can be found in figure 3*a* (MCF-10A, MCF-7, MDA-MB-231) and in the electronic supplementary material, figure S3.

**Figure 4.**
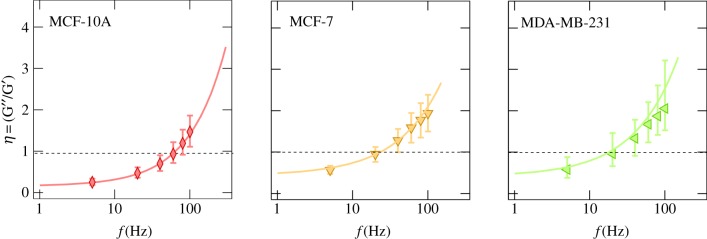
Loss tangent *η* = *G*″*/G*′ of the human breast cell lines MCF-10A, MCF-7 and MDA-MB-231 as a function of frequency. Continuous lines represent results of fitting the parameters of the power-law structural damping model to the experimental data.

**Figure 5.**
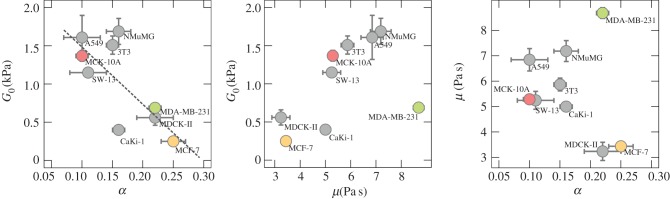
Correlation plots of the parameters *G*_0_, *α* and *μ* for all cell lines. A correlation between *G*_0_ and the power-law exponent *α* is found for human breast cell lines (coloured symbols connected by dotted line).

First, we focus on the cell lines MCF-10A, MCF-7 and MDA-MB-231, all from the human mammary gland but with different metastatic potential. Figure 3*b* compiles the results of the microrheological investigation. Rheological data of the other six cell lines are shown in the electronic supplementary material, figure S4. The presented data are computed from at least two force maps per cell line with a resolution of 32 × 32 pixels. In general, the complex shear modulus of all cell lines followed the typical frequency dependence found for many other cell types, including neutrophils, airway smooth muscle cells, bronchial epithelial cells or pulmonary macrophages with different microrheological methods [14,15]. *G*′ increases with frequency following a weak power law with exponents *α* ranging from 0.10 to 0.25 (table 1), while *G*″ exhibits lower values compared with *G*′ in the low-frequency regime (less than 50 Hz). In this regime, the cells show a more solid-like behaviour as the loss tangent (*η* = *G*″*/G*′) does not exceed 1 (see also figure 4 and electronic supplementary material, figure S5 for all cell lines). However, the high-frequency domain is dominated by *G*″, indicating that the cells at high frequencies behave more like a viscous liquid (*η* > 1). An attempt to explain this power-law behaviour in the microrheological spectra of living cells has been made by Kollmannsberger & Fabry [27]. By describing the cell as an active soft glassy material, some rheological features can be assigned to cytoskeletal organization and remodelling. The model is based on the soft glassy rheology model first described by Sollich [28], and assumes that the cytoskeleton of the cell consists of many disordered elements, which are held together by weak attractive forces between neighbouring elements trapping the elements in energy wells. These weak interactions allow the elements to occasionally jump between the potential wells. A large distribution of energy well depth leads to a scale-free (power-law) behaviour of the lifetime distribution and thus results in a power-law rheological behaviour. The power-law coefficient corresponds to the effective temperature of the material [15]. The material is at the thermal equilibrium if the power-law coefficient becomes 0. The model of active soft glassy rheology also predicts power-law structural damping behaviour, at least at intermediate time scales. The rheological data of all cell lines could be well described by this model (solid lines in figure 3*b* and electronic supplementary material, figure S4). The obtained fitting parameters of all cell lines according to electronic supplementary material, equation (S8) are summarized in table 1. We found that the measured values of *G*′ (storage modulus)*, G*_0_ (scaling factor), *α* (power-law exponent) or *μ* (Newton viscosity term) for all cell lines do not show a clear correlation to the malignancy of the cell line. MCF-7, CaKi-1 and MDA-MB-231 cells, three malignant cancer cell lines, show the lowest *G′*-values at all frequencies, followed by epithelial MDCKII cells with values slightly higher than those of MDA-MB231 cells. Furthermore, malignant A549, NIH 3T3, MCF-10A and NMuMG cells display the highest storage modules. However, considering cells only from one organ (human mammary gland), we can confirm that benign cells are stiffer than malignant ones (figure 3; electronic supplementary material, figure S4). Apart from the obvious stiffness differences that have also been reported by others, we found that the power-law exponent of benign MCF-10A cells is considerably smaller (figure 5). Other than that, no correlation between the various parameters obtained from fitting electronic supplementary material, equation (S8) to the rheological spectra was found (figure 5).

**Table 1.** Fitting parameters *G*_0_, *α* and *μ* of the power-law structural damping model (see the electronic supplementary material, equation (S8)) applied to the used cell lines with corresponding standard error. *G*_0_ denotes the shear modulus at zero frequency, *μ* the viscosity and *α* is the power-law coefficient.

	*G*_0_/kPa	*α*	*μ*/Pa × s
NIH 3T3	1.51 ± 0.12	0.15 ± 0.01	5.87 ± 0.25
MDCKII	0.56 ± 0.10	0.22 ± 0.03	3.24 ± 0.36
MCF-10A	1.37 ± 0.07	0.10 ± 0.01	5.30 ± 0.1
NMuMG	1.69 ± 0.17	0.16 ± 0.02	7.19 ± 0.41
A549	1.61 ± 0.29	0.10 ± 0.03	6.85 ± 0.44
MDA-MB-231	0.69 ± 0.06	0.22 ± 0.01	8.69 ± 0.21
CaKi-1	0.40 ± 0.02	0.16 ± 0.01	5.00 ± 0.04
MCF-7	0.25 ± 0.02	0.25 ± 0.02	3.44 ± 0.10
SW13	1.15 ± 0.17	0.11 ± 0.03	5.25 ± 0.35

Several groups with divergent findings have extensively investigated the effect of malignant transformation of cells on cellular mechanics. Lekka *et al*. [29], for instance, investigated the elastic response of normal and cancerous human bladder cell lines using the AFM. They found that cancerous cells exhibit elastic moduli that are one order of magnitude lower compared with normal cells. Guck *et al*. [30], who measured the deformability of non-cancerous and cancerous human mammary epithelial cells with a microfluidic optical stretcher, have observed a similar trend. In addition, the cancerous cells were found to be more deformable than the corresponding benign cells. Treatment of the cancer cells with 12-*O*-tetradecanoylphorbol-13-acetate, a tumour-promoting drug, additionally enhanced the deformability of the cancer cells. It was found that cancerous MCF-7 cells are more deformable than benign MCF-10, and that the metastatic modified MCF-7 cells are even more deformable than MCF-7 cells [3]. The increased deformability of cancer cells with modified metastatic competence is accompanied by a reduction in structural strength opposed to non-metastatic cancer cells. This reduction in elastic rigidity might be attributed to a decrease in F-actin concentration by as much as 30% at small strain levels, while at larger strain reorganization in the molecular architecture of keratins might be responsible.

**Figure 6.**
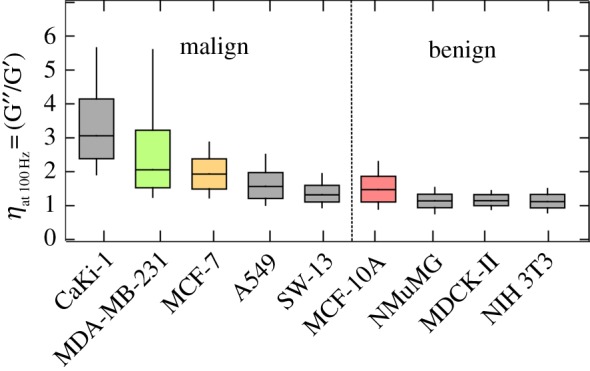
Loss tangent *η* of all cell lines with different metastatic potential computed at an oscillation frequency of 100 Hz (two force maps, corresponding to 5–20 cells, depending on cell size).

On the other hand, Zhang *et al*. [6] report on a stiffening of hepatocellular carcinoma cells compared with benign hepatocytes. These contradictory results mirror the difficulty of categorizing the malignancy of cancer cells according to their softness (i.e. Young's modulus). Nevertheless, the malignant transformation of benign cells into metastatic cancer cells results from a change in the protein expression pattern of the cell, which is accompanied by a reorganization of the cytoskeleton and a change in the adhesive properties of the cells [3,31]. Taking the histological origin of the cells into account, we can confirm the trend towards softer cancer cells compared with stiffer benign cells in our measurements. The benign epithelial cell lines NMuMG, MCF-10A and MDCKII, as well as the very stiff fibroblasts, show higher values for *G*′ compared with the corresponding malignant cancer cell lines from the same organ. However, A549 cells do not follow a potentially universal trend of low elastic modules observed for malignant cells. Problems of using the elastic modulus for the cancer cell detection have been discussed recently by Lekka & Laidler [32]. By contrast, the loss tangent *η* as a model-independent parameter (as it is solely described by the phase shift between excitation of the cantilever and its response) does not assume a particular geometry of indenter or cell. Thus, it is independent of the indentation depth and details of contact mechanics providing a quantitative measure of the overall rheological behaviour of the sample. Figure 6 shows the loss tangent *η* determined at an oscillation frequency of 100 Hz for all cell lines. The complete frequency dependence of the loss tangent is depicted in figure 4 for the three breast cell lines. Electronic supplementary material, figure S6 compares the benign cell line MCF-10A with the malign one MDA-MB-231 at the full frequency range, demonstrating that the non-tumorigenic cell line possesses a smaller loss tangent at all frequencies. At 100 Hz oscillation frequency, both epithelial cell lines (MDCKII and NMuMG cells) and the fibroblasts (NIH 3T3 cells) exhibit a loss tangent *η* of approximately 1.1 with a narrow distribution. All cancer cell lines have significantly higher median values of *η*, ranging from 1.3 in the case of A549 cell up to 2 and 3 in the case of MDA-MB231 cells and CaKi-1 cells, respectively. The effect is also accompanied by a broadening of the distribution. The elevated values of *η* can be interpreted as a more fluid-like behaviour of the malign cancer cells compared with the benign cell lines. We interpret this effect by the concomitant migratory behaviour of cancer cells. To form metastases, cancer cells have to detach from their primary tumour and invade the parenchyma and the vasculature [33]. During this process, the cancer cells have to undergo large deformations (i.e. during intra- and extravasation), while they are ‘squeezing’ themselves through endothelial cell layers. Hence, a more fluid-like behaviour of the cells facilitates this process, which is critical for the formation of metastasis in tissues far away from the primary tumour. While a large loss tangent identifies a more fluid-like behaviour it does not automatically mean that the stiffness of the cell is low. It just means that viscosity dominates over elasticity.

## 6. Conclusion

The mechanical properties of cells are governed by a multitude of passive and active elements, and are far from being understood in their full complexity. Simple contact mechanical models that explain rheology over a wide range of time and length scales cannot capture all the features of the complex cellular structure. Moreover, the answer of the cells to external deformation on longer time scales is influenced by biochemical and genetic adaption to the mechanical stimulus, rendering the overall response even more intricate. However, it is indisputable that cancer cells and benign cells display different elastic properties, which might serve as a diagnostic tool to readily identify malignant phenotypes by their mechanical signature and response to mechanical stimulation.

Here, we propose a model-free approach based on monitoring the loss tangent that essentially represents the ratio of loss modulus to storage modulus of the probed cell, and therefore quantifies energy dissipation upon deformation at different frequencies. We found that cancer cells show a substantially larger loss tangent than the benign phenotype, which means that the cells behave more like fluid at smaller time scales (larger frequency).

## Supplementary Material

Data Supplement

## References

[1] Hanahan D Weinberg RA 2011 Hallmarks of cancer: the next generation. Cell 144, 646–674. (doi:10.1016/J.Cell.2011.02.013) 21376230 10.1016/j.cell.2011.02.013

[2] Olson MF Sahai E 2009 The actin cytoskeleton in cancer cell motility. Clin. Exp. Metastasis 26, 273–287. (doi:10.1007/S10585-008-9174-2) 18498004 10.1007/s10585-008-9174-2

[3] Suresh S Spatz J Mills JP Micoulet A Dao M Lim CT Beil M Seufferlein T 2005 Connections between single-cell biomechanics and human disease states: gastrointestinal cancer and malaria. Acta Biomater. 1, 15–30. (doi:10.1016/J.Actbio.2004.09.001) 16701777 10.1016/j.actbio.2004.09.001

[4] Agus DB 2013 A physical sciences network characterization of non-tumorigenic and metastatic cells. Sci. Rep. UK 3, 1449 (doi:10.1038/Srep01449) 10.1038/srep01449 PMC3636513 23618955

[5] Rosenbluth MJ Lam WA Fletcher DA 2006 Force microscopy of nonadherent cells: a comparison of leukemia cell deformability. Biophys. J. 90, 2994–3003. (doi:10.1529/Biophysj.105.067496) 16443660 10.1529/biophysj.105.067496 PMC1414579

[6] Zhang G Long M Wu ZZ Yu WQ 2002 Mechanical properties of hepatocellular carcinoma cells. World J. Gastroenterol. 8, 243–246. 11925600 10.3748/wjg.v8.i2.243 PMC4658359

[7] Mizuno D Tardin C Schmidt CF MacKintosh FC 2007 Nonequilibrium mechanics of active cytoskeletal networks. Science 315, 370–373. (doi:10.1126/Science.1134404) 17234946 10.1126/science.1134404

[8] Pullarkat PA Fernandez PA Ott A 2007 Rheological properties of the eukaryotic cell cytoskeleton. Phys. Rep. Rev. Sect. Phys. Lett. 449, 29–53. (doi:10.1016/j.physrep.2007.03.002)

[9] Binnig G Quate CF Gerber C 1986 Atomic force microscope. Phys. Rev. Lett. 56, 930–933. (doi:10.1103/Physrevlett.56.930) 10033323 10.1103/PhysRevLett.56.930

[10] Kuznetsova TG Starodubtseva MN Yegorenkov NI Chizhik SA Zhdanov RI 2007 Atomic force microscopy probing of cell elasticity. Micron 38, 824–833. (doi:10.1016/J.Micron.2007.06.011) 17709250 10.1016/j.micron.2007.06.011

[11] Shroff SG Saner DR Lal R 1995 Dynamic micromechanical properties of cultured rat atrial myocytes measured by atomic-force microscopy. Am. J. Physiol. 269, C286–C292. 7631757 10.1152/ajpcell.1995.269.1.C286

[12] Alcaraz J Buscemi L Grabulosa M Trepat X Fabry B Farre R Navajas D 2003 Microrheology of human lung epithelial cells measured by atomic force microscopy. Biophys. J. 84, 2071–2079. (doi:10.1016/S0006-3495(03)75014-0) 12609908 10.1016/S0006-3495(03)75014-0 PMC1302775

[13] Mahaffy RE Shih CK MacKintosh FC Kas J 2000 Scanning probe-based frequency-dependent microrheology of polymer gels and biological cells. Phys. Rev. Lett. 85, 880–883. (doi:10.1103/Physrevlett.85.880) 10991422 10.1103/PhysRevLett.85.880

[14] Smith BA Tolloczko B Martin JG Grutter P 2005 Probing the viscoelastic behavior of cultured airway smooth muscle cells with atomic force microscopy: stiffening induced by contractile agonist. Biophys. J. 88, 2994–3007. (doi:10.1529/Biophysj.104.046649) 15665124 10.1529/biophysj.104.046649 PMC1305393

[15] Fabry B Maksym GN Butler JP Glogauer M Navajas D Fredberg JJ 2001 Scaling the microrheology of living cells. Phys. Rev. Lett. 87, Article no. 148102 (doi:10.1103/Physrevlett.87.148102) 10.1103/PhysRevLett.87.148102 11580676

[16] Bilodeau GG 1992 Regular pyramid punch problem. J. Appl. Mech. 59, 519–523. (doi:10.1115/1.2893754)

[17] Landau LD Lifshitz EM Kosevitch AM Pitaevskiĭ LP 1986 Theory of elasticity. Burlington, MA: Butterworth-Heinemann.

[18] Clark AG Paluch E 2011 Mechanics and regulation of cell shape during the cell cycle. Results Probl. Cell D 53, 31–73. (doi:10.1007/978-3-642-19065-0_3) 10.1007/978-3-642-19065-0_3 21630140

[19] Schroede Te 1972 Contractile ring. 2. Determining its brief existence, volumetric changes, and vital role in cleaving *Arbacia* eggs. J. Cell Biol. 53, 419 (doi:10.1083/Jcb.53.2.419) 5063470 10.1083/jcb.53.2.419 PMC2108733

[20] Gavara N Chadwick RS 2012 Determination of the elastic moduli of thin samples and adherent cells using conical atomic force microscope tips. Nat. Nanotechnol. 7, 733–736. (doi:10.1038/Nnano.2012.163) 23023646 10.1038/nnano.2012.163 PMC3492504

[21] Dukes JD Whitley P Chalmers AD 2011 The MDCK variety pack: choosing the right strain. BMC Cell Biol. 12, article no. 43 (doi:10.1186/1471-2121-12-43) 10.1186/1471-2121-12-43 PMC3209442 21982418

[22] Shafie SM Liotta LA 1980 Formation of metastasis by human-breast carcinoma-cells (Mcf-7) in nude-mice. Cancer Lett. 11, 81–87. (doi:10.1016/0304-3835(80)90097-X) 6450636 10.1016/0304-3835(80)90097-x

[23] Fujimoto E 2005 Connexin32 as a tumor suppressor gene in a metastatic renal cell carcinoma cell line. Oncogene 24, 3684–3690. (doi:10.1038/Sj.Onc.1208430) 15782139 10.1038/sj.onc.1208430

[24] Hiraga T Williams PJ Mundy GR Yoneda T 2001 The bisphosphonate ibandronate promotes apoptosis in MDA-MB-231 human breast cancer cells in bone metastases. Cancer Res. 61, 4418–4424. 11389070

[25] Sussan TE Pletcher MT Murakami Y Reeves RH 2005 Tumor suppressor in lung cancer I (TSLCI) alters tumorigenic growth properties and gene expression. Mol. Cancer 4, 28 (doi:10.1186/1476-4598-4-28) 10.1186/1476-4598-4-28 PMC1208945 16083501

[26] Ziegler CG 2009 Expression of neuropeptide hormone receptors in human adrenal tumors and cell lines: antiproliferative effects of peptide analogues. Proc. Natl Acad. Sci. USA 106, 15 879–15 884. (doi:10.1073/Pnas.0907843106) 10.1073/pnas.0907843106 PMC2733863 19717419

[27] Kollmannsberger P Fabry B 2009 Active soft glassy rheology of adherent cells. Soft Matter 5, 1771–1774. (doi:10.1039/B820228a)

[28] Sollich P 1998 Rheological constitutive equation for a model of soft glassy materials. Phys. Rev. E 58, 738–759. (doi:10.1103/Physreve.58.738)

[29] Lekka M Laidler P Gil D Lekki J Stachura Z Hrynkiewicz AZ 1999 Elasticity of normal and cancerous human bladder cells studied by scanning force microscopy. Eur. Biophys. J. Biophy. 28, 312–316. (doi:10.1007/S002490050213) 10.1007/s002490050213 10394623

[30] Guck J 2005 Optical deformability as an inherent cell marker for testing malignant transformation and metastatic competence. Biophys. J. 88, 3689–3698. (doi:10.1529/Biophysj.104.045476) 15722433 10.1529/biophysj.104.045476 PMC1305515

[31] Cross SE Jin YS Rao J Gimzewski JK 2007 Nanomechanical analysis of cells from cancer patients. Nat. Nanotechnol. 2, 780–783. (doi:10.1038/Nnano.2007.388) 18654431 10.1038/nnano.2007.388

[32] Lekka M Laidler P 2009 Applicability of AFM in cancer detection. Nat. Nanotechnol. 4, 72 (doi:10.1038/nnano.2009.004) 19197298 10.1038/nnano.2009.004

[33] Kumar S Weaver V 2009 Mechanics, malignancy, and metastasis: the force journey of a tumor cell. Cancer Metastasis Rev. 28, 113–127. (doi:10.1007/S10555-008-9173-4) 19153673 10.1007/s10555-008-9173-4 PMC2658728

