# Supplementary material for: Atomic force microscopy-based microrheology reveals significant differences in the viscoelastic response between malign and benign cell lines
Source: Open Biol. 2014 May 21;4(5):140046. doi: 10.1098/rsob.140046 (PMC4042852; doi:10.1098/rsob.140046)
Supplement: Data Supplement [file rsob140046supp1.pdf]

Supplementary Information

AFM-based microrheology reveals significant differences in the viscoelastic response between malign and benign cell lines

Jan Rother[a], Helen Nöding[a], Ingo Mey[b] and Andreas Janshoff[a]*

[a] *Institute of Physical Chemistry, Tammannstr. 6, 37077 Göttingen, Germany, Tel: 049 0551 39 10633; E-mail: ajansho@gwdg.de*

[b] *Institute of Organic and Biomolecular Chemistry, Tammannstr. 2, 37077 Göttingen, Germany, Tel: 049 0551 39 10633;*

Procedure to carry out microrheology with an AFM

The general procedure to carry out microrheological experiments with the AFM relies on the work of Alcaraz *et al.*[1] The complex shear modulus $G*$ is given by

$$G^*(\omega) = G'(\omega) + iG''(\omega) = \frac{1-\nu}{3\delta_0 \tan(\varphi)} \frac{F(\omega)}{\delta(\omega)} \qquad \text{(S1)}$$

and the ratio between the force oscillation $F(\omega)$ and the separation oscillation $\delta(\omega)$ depends on the measured amplitudes A_F of the force signal and A_δ of the indentation signal at a given frequency ω:

$$\frac{F(\omega)}{\delta(\omega)} = \frac{A_F(\omega)}{A_\delta(\omega)} e^{i(\varphi_{F(\omega)} - \varphi_{\delta(\omega)})}, \qquad \text{(S2)}$$

where φ_F *and* φ_δ are the phase shifts of the force oscillation and the separation oscillation, respectively. The complex shear modulus consists of a real part G' called storage modulus, which accounts for the energy stored in the system and an imaginary part G'' called loss modulus, which accounts for energy dissipated in the sample. The ratio between loss modulus and storage modulus is called loss tangent η and is given by the tangent of the phase shift $\Delta\varphi$ between the two sinusoidal signals of $F(\omega)$ and $\delta(\omega)$ (see also Supplementary Fig. S2):

$$\eta = \tan\left(\varphi_{F(\omega)} - \varphi_{\delta(\omega)}\right) = \tan\left(\Delta\varphi\right) \qquad \text{(S3)}$$

The loss tangent is a model independent parameter, which does not rely on geometrical factors. For a pure elastic solid η is zero, while it approaches infinity for a purely viscous sample.

As the cantilever is always in contact with the viscous medium during the microrheological experiment, the measured force is the sum of the hydrodynamic force and the force response of the sample. To correct for the hydrodynamic force acting on the cantilever during oscillation, we used a method introduced by Alcaraz *et al.* [2] The force response ΔF_{HDD} of the cantilever to small amplitude oscillations Δz was measured for several oscillation frequencies as a function of the tip-sample separation h (Fig. S1A). Before correction of the hydrodynamic drag, the deviation from 90° phase shift in a viscous environment φ_{lag} was compensated for every frequency f in each measurement (see Fig. S2). Afterwards, the transfer function $H_D{}^*$, given by

$$H_D{}^*(f) = H_D{}' + iH_D{}''(f) = \frac{kH_a(f)}{k - H_a(f)} \text{ with} \qquad \text{(S4)}$$

$$H_a(f) = \frac{A_{\Delta F}}{A_{\Delta z}} \frac{F(\omega)}{\delta(\omega)} e^{i(\varphi_{F(\omega)} - \varphi_{\delta(\omega)} - \varphi_{lag})}, \qquad \text{(S5)}$$

was determined for every frequency at different tip-sample distances h by fitting a sine wave with the amplitude A and the phase shift φ to Δz and ΔF_{HDD} (Fig. S1B). Here, k is the spring constant of the cantilever and $A_{\Delta F}$, $A_{\Delta z}$, $\varphi_{\Delta F}$ *and* $\varphi_{\Delta z}$ are the amplitudes and the phases of ΔF_{HDD} and Δz.

Fig. S1C shows an example for the frequency dependence of the transfer function H_D^* at a fixed tip-sample separation. The imaginary part H_d'' increases linearly with oscillation frequency, while the real part can be neglected. Linear fitting of H_d'' provides the drag coefficient $b(h)$ as the slope m of the fit is given by $m=2\pi b(h)$.

The drag coefficient is determined at tip-sample separations ranging from *200* nm to *3500* nm. The dependency of the drag coefficient $b(h)$ from the tip-sample separation is shown in Fig. S1D. At low tip-substrate distances, a higher drag coefficient is observed. The drag coefficient as a function of the tip-sample separation is fitted with the scaled spherical model of the cantilever

$$b(h) = \frac{6\pi\eta {a_{eff}}^2}{h+h_{eff}} \qquad \text{(S6)}$$

with the dynamic viscosity of the medium η and the two fitting parameters a_{eff} and h_{eff} that account for the effecting cantilever geometry. The drag coefficient at zero tip sample separation $b(h_0)$ was extrapolated from that fit. This drag coefficient $b(h_0)$ was used in the microrheological experiment to correct for the hydrodynamic drag in the imaginary part of G^*

$$G^*(\omega) = G'(\omega) + iG''(\omega) = \frac{1-\nu}{3\delta_0 \tan(\theta)}\left(\frac{F(\omega)}{\delta(\omega)} - i\omega b(h_0)\right). \quad \text{(S7)}$$

The obtained data for G* were fitted using the power law structural damping model used by Alcaraz *et al.*:[1]

$$G^* = G_0\left(1 + i\,tan\left(\alpha \cdot \frac{\pi}{2}\right)\right)\left(\frac{\omega}{\omega_0}\right)^{\alpha} + i\mu\omega \qquad \text{(S8)}$$

G_0 is a scaling factor, α is the power law exponent and μ denotes the viscosity of the sample. Fig.S3 confirms that the loss tangent η does not depend on the indentation depth δ in contrast to all other parameters due to shortcoming of the contact model.

1. J. Alcaraz, L. Buscemi, M. Grabulosa, X. Trepat, B. Fabry, R. Farre, D. Navajas, Microrheology of human lung epithelial cells measured by atomic force microscopy. *Biophys. J.* 2003, *84*. 2071-2079.

2. J. Alcaraz, L. Buscemi, M. Puig-de-Morales, J. Colchero, A. Baro, D. Navajas, Correction of microrheological measurements of soft samples with atomic force microscopy for the hydrodynamic drag on the cantilever. *Langmuir* 2002, *18*. 716-721, DOI: Doi 10.1021/La0110850.

Supplementary Information Figures

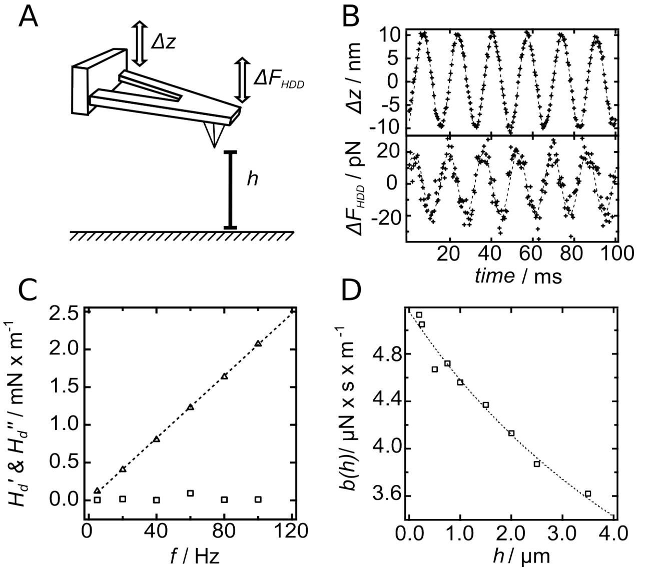

Fig. S1 **A** Scheme illustrating the correction of force data for hydrodynamic drag. The triangular cantilever is kept at a height *h* above the surface and oscillates with a z-displacement of Δz at its basis. The force response ΔF_{HDD} is determined over the deflection of the cantilever and its spring constant. **B** Δz and ΔF_{HDD} as a function of time. Dots are the measured data; the dashed line shows the fit of both signals. **C** Real part of the transfer function H_D' (squares) and imaginary part H_D'' (triangles) as a function of the oscillation frequency f at a fixed tip surface distance h. The dashed line shows linear fit of H_D'' with the slope $2\pi b(h)$. **D** Drag coefficient $b(h)$ (squares) as a function of the tip-sample separation h. The data were fitted using a scaled spherical model (dashed line). Extrapolation of the fit to $h = 0$ nm delivers the drag coefficient $b(h_0)$ used for the hydrodynamic drag correction.

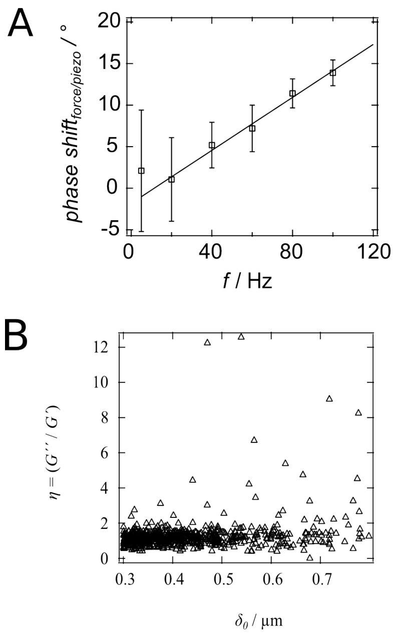

Fig. S2 A Example of deviation from 90° phase shift of Δz and ΔF_{HDD} (squares) as a function of the oscillation frequency measured in viscous medium. Data were fitted using a linear fit applied to all data above an oscillation frequency of 10 Hz. Deviations of more than 20% from 90° were also not taken into account. **B** Loss tangent of NMuMG cells computed for 100 Hz oscillation frequency as a function of the indentation depth.

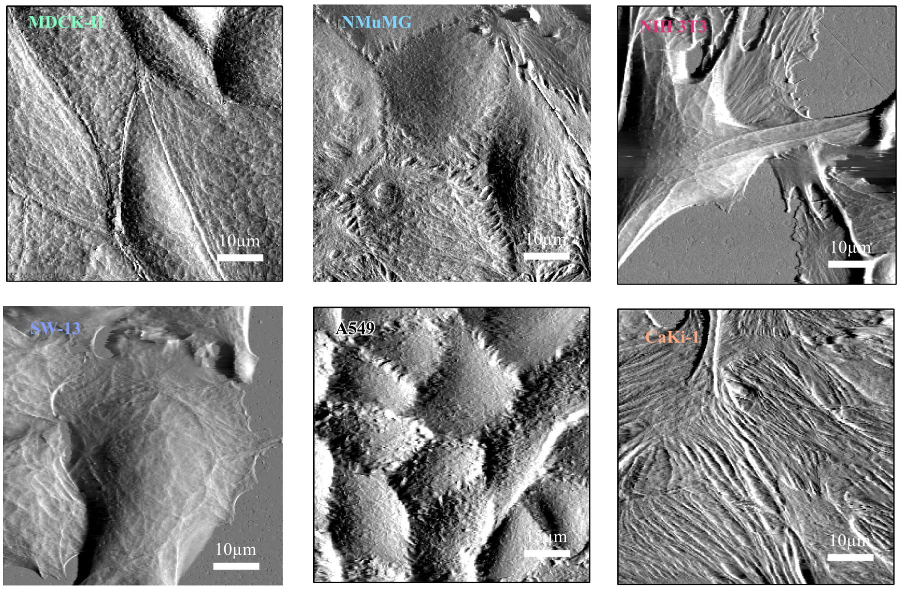

Fig. S3 AFM images (deflection) of cell lines used in this study.

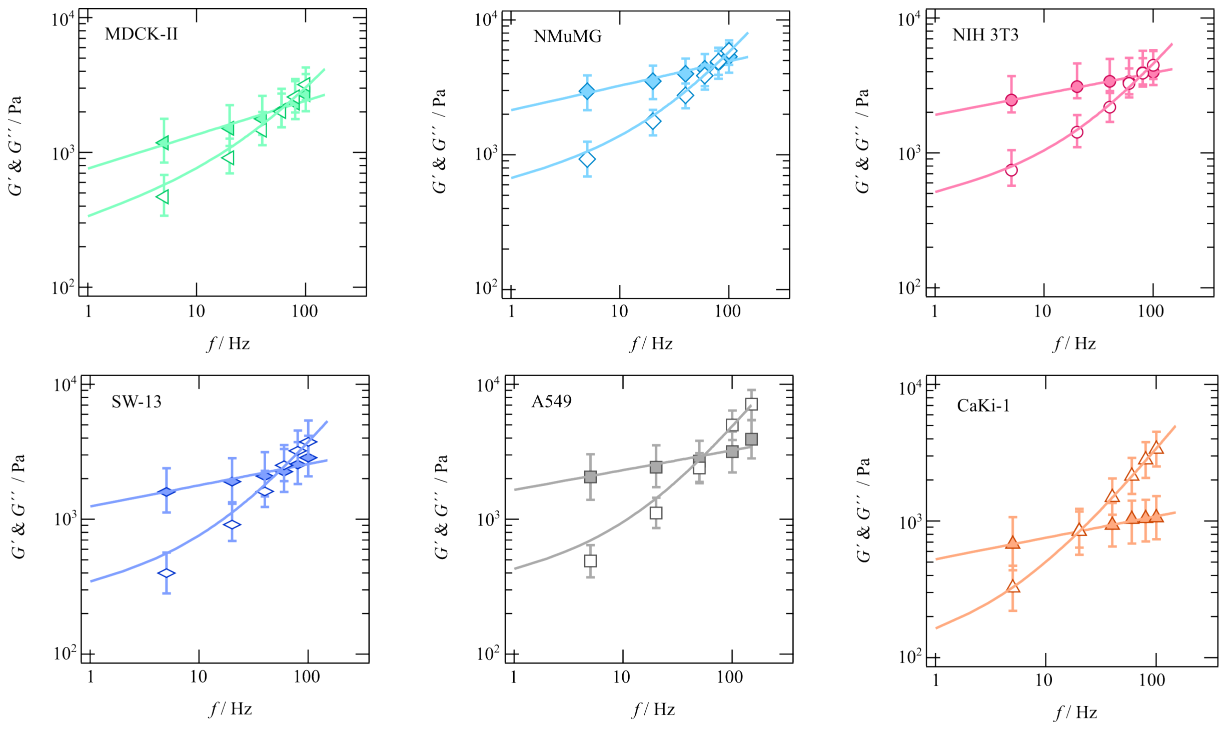

Fig. S4 Median values of the storage modulus G' (closed symbols) and loss modulus G'' (open symbols) of cell lines with different metastatic potential as a function of the oscillation frequency (2 force maps, >10 cells). MDCKII and NMuMG cells are non-metastatic immortalized epithelial cells, while NIH 3T3 are benign mesenchymal cells. A549, SW13 and the CaKi-1 cells are metastatic cancer cells. The data of the complex shear modulus were fitted using the power-law structural damping model (dashed line).

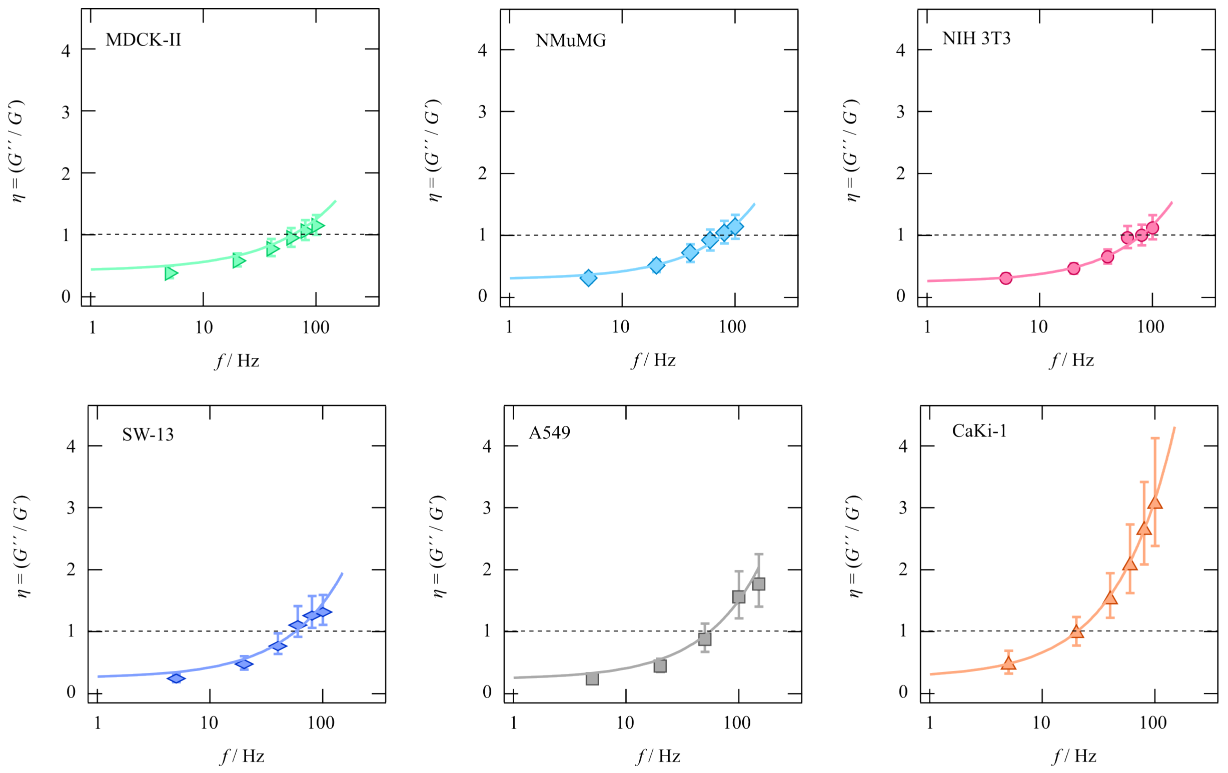

Fig. S5 Loss tangent η of cell lines MDCKII, NMuMG, NIH 3T3, SW13, A549 and CaKi-1 cells with different metastatic potential (2 force maps, >10 cells). Dashed line shows course of loss tangent determined from the power law structural damping model.

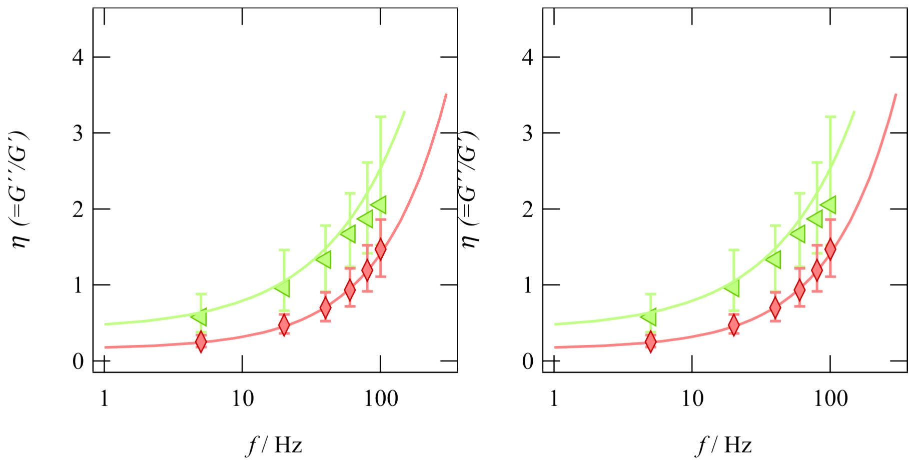

Fig. S6 Loss tangent η of benign MCF-10A cells (red diamonds) in comparison to malignant MDA-MD-231 cells (green triangles). Continuous lines are the correspond fits according to the structural damping model.
